# Self-reported reasons for reducing or stopping antidepressant medications in primary care: thematic analysis of the *diamond* longitudinal study

**DOI:** 10.1017/S1463423623000038

**Published:** 2023-02-27

**Authors:** Amy Coe, Jane Gunn, Susan Fletcher, Elizabeth Murray, Catherine Kaylor-Hughes

**Affiliations:** 1 Department of General Practice, The University of Melbourne, Melbourne, VIC, 3004, Australia; 2 Research Department of Primary Care and Population Health, University College, London, NW3 2PF, UK

**Keywords:** depression, general practice, antidepressants, deprescribing

## Abstract

**Background:**

Current treatment guidelines advise that the deprescribing of antidepressants should occur around 6 months post-remission of symptoms. However, this is not routinely occurring in clinical practice, with between 30% and 50% of antidepressant users potentially continuing treatment with no clinical benefit. To support patients to deprescribe antidepressant treatment when clinically appropriate, it is important to understand what is important to patients when making the decision to reduce or cease antidepressants in a naturalistic setting.

**Aim:**

The current study aimed to describe the self-reported reasons primary care patients have for reducing or stopping their antidepressant medication.

**Methods:**

Three hundred and seven participants in the *diamond* longitudinal study reported taking an SSRI/SNRI over the life of the study. Of the 307, 179 reported stopping or tapering their antidepressant during computer-assisted telephone interviews and provided a reason for doing so. A collective case study approach was used to collate the reasons for stopping or tapering.

**Findings:**

Reflexive thematic analysis of patient-reported factors revealed five overarching themes; 1. Depression; 2. Medication; 3. Healthcare system; 4. Psychosocial, and; 5. Financial. These findings are used to inform suggestions for the development and implementation of antidepressant deprescribing discussions in clinical practice.

## Introduction

Antidepressant medications are the first-line treatment for depression considered to be more severe (moderate to severe depression) (Iacobucci, 2019; Malhi *et al*., 2021; National Institute for Health and Care Excellence (NICE), 2022). In Australia, prescriptions for selective serotonin reuptake inhibitors (SSRIs) and serotonin norepinephrine reuptake inhibitors (SNRIs) have doubled in the past 10 years (Australian Institute of Health and Welfare, 2022 and 2013) with similar increases being seen globally (OECD, 2019). Evidence suggests that much of the increase in antidepressant use can be accounted for by long-term use (>2 years) (Mojtabai and Olfson, 2013; Verhaak *et al*., 2019) rather than an increase in the incidence and prevalence of depression (Munoz-Arroyo *et al*., 2006). For some patients, longer-term antidepressant use is recommended (Malhi *et al*., 2021; National Institute for Health and Care Excellence (NICE), 2022); however, it is estimated that between 30% and 50% of patients are continuing treatment without experiencing any clinical benefit (Ambresin *et al*., 2015; Cruickshank *et al*., 2008; Davidson *et al*., 2020).

In line with growing concern over the increase in long-term antidepressant use, treatment guidelines from the United Kingdom (UK) and Australia and New Zealand were recently updated to reflect recommendations that gradual dose reduction and eventual cessation (deprescribing) may occur at around 6 months after remission (Malhi *et al*., 2021; National Institute for Health and Care Excellence (NICE), 2022). Most (87%) (Australian Institute of Health and Welfare, 2022) antidepressants are prescribed by general practitioners (GPs), placing them in the best position to also deprescribe. However, patient and GP barriers including a lack of guidance to deprescribe (Bowers *et al*., 2019; Sørensen *et al*., 2022; Van Leeuwen *et al*., 2021) (Maund *et al*., 2019a; Sørensen *et al*., 2022), fear of deprescribing (Leydon *et al*., 2007; Maund *et al*., 2019a; Verbeek-Heida and Mathot, 2006) and clinical inertia (Henke *et al*., 2009) have caused a lack of routine deprescribing in clinical practice. A recent scoping review (Coe *et al*., 2021) suggests that there are a variety of activities available to encourage GP-led deprescribing; however, there is a need to strengthen the knowledge about how to best support patients to cease medication when clinically appropriate (Todd *et al*., 2018).

Studies focusing on patient experiences of taking antidepressants show that up to 60% of antidepressants users attempt reducing or stopping their medications (Read *et al*., 2019) even without professional guidance. Respondents in a community mental health survey in the UK (Read *et al*., 2019) reported reducing or stopping if they felt they no longer required the medication, had experienced side effects or had concerns about long-term use. Of the 257 participants, just 9% (*n* = 24) reported stopping due to GP or psychiatrist advice. Similar sentiments have been found in qualitative studies exploring patient experiences of antidepressants in primary care. Antidepressant users in the UK (Leydon *et al*., 2007) reported that feeling well, having GP support or experiencing uncertainty about the effectiveness of antidepressants were factors for reducing or stopping. For a sample of Dutch antidepressant users (Verbeek-Heida and Mathot, 2006), fear of addiction, stigma and advice from GPs greatly influenced the decision to stop treatment. A recent Belgian study found guidance by GPs to be a strong facilitator of deprescribing (Van Leeuwen *et al*., 2022). Despite these user experience studies, qualitative primary care studies reporting reasons that patients reduce or stop their antidepressant treatment are still limited.

Therefore, the aim of the current study was to describe the reasons why Australian general practice patients reduce or stop their SSRI or SNRI medication using qualitative data collected as part of a 10-year longitudinal study.

## Method

### The diamond study design

The Diagnosis, Management and Outcomes of Depression (*diamond*) longitudinal study ran between 2005 and 2015. Details of the *diamond* study methods, participants and outcomes have been published elsewhere (Ambresin *et al*., 2015; Davidson *et al*., 2020; Gunn *et al*., 2008). Briefly, the *diamond* study recruited 789 patients with depressive symptoms from 30 randomly selected general practices across Victoria, Australia. Potential participants were identified using the following eligibility criteria: 1. Aged between 18 and 75 years; 2. Proficient in English; 3. Not terminally ill or residing in a nursing home, and; 4. Scored ≥16 on the Centre for Epidemiologic Studies Depression Scale (CES-D) (Radloff, 1977), indicating presence of depressive symptoms. Postal surveys were completed at enrolment with follow-up surveys every three months from baseline in the first year, and annually after that. In addition, computer-assisted telephone interviews (CATIs) were conducted annually by trained research assistants.

Slight variations in wording occurred between questions asked in earlier CATIs and later CATIs. This was due to revisions made by the *diamond* research team to account for the time that had passed since the previous CATI (e.g., ‘have you ever stopped’ (12-month CATI) was changed to ‘in the past 12 months have you ever stopped’ (subsequent CATIs)). Some questions were revised to improve clarity and elicit a more considered response from participants. See Appendix A for original and revised questions used in the current study. AC read and re-read all responses to the original and revised questions and found no discrepancies in the meaning or depth of responses.

### Data collection

Qualitative data from 307 antidepressant users who completed at least one CATI and demographic information from the baseline postal survey are reported in this study. CATIs followed a structured interview guide developed by an interdisciplinary team of researchers (Gunn *et al*., 2008) exploring participant experiences and views on depression, depression care, social support and attitudes towards depression and were developed by the aforementioned research team. At each CATI time point, participants were asked a series of quantitative and qualitative questions about the medications they were taking for their mental health in the 12 months leading up to the CATI. These questions included medication name, medication dosage, profession of prescribing clinician, and length of medication use, whether the participant had forgotten to take their medication and whether they had stopped their medication and why. For questions where a ‘no’ response was indicated, skip logic was applied; therefore, every participant may not have been asked each question.

Participant responses to questions were typed verbatim into a purpose-built database, and all CATIs from the fourth time point onwards were digitally recorded for quality assessment. CATIs at each time point were structured the same, except for the first interview which collected baseline data and did not include questions on antidepressant cessation; as such, the baseline interview was excluded from the current study.

### Data collation

A deidentified dataset of 307 participants who had reported taking SSRIs or SNRIs during at least one CATI time point was created by the *diamond* Data Manager and provided to AC. This dataset included participant responses to two primary questions which explicitly asked why they stopped their antidepressant medication in the last 12 months (see questions 1 and 2 in Table 1).

**Table 1.** Participant CATI questions pertaining to reducing or stopping antidepressants

	Question
1	In the past 12 months, have you stopped taking (antidepressant medication name) even if only for a short time? What made you stop taking (antidepressant medication name)?
2	Are you for some reason not taking any (other) medicines which have been prescribed for you (that you are supposed to be taking) for your emotional or physical well-being? Why are not taking them?
3	During the past 12 months, of everything you have done or tried for depression, stress or worries, what has been the least helpful?
4	Can you describe how you have been feeling since we last spoke about 12 months ago?

To identify if any references to reducing or stopping antidepressant medications lay outside responses to the two primary questions, AC conducted a 10% (*n* = 14) check of available digital CATI recordings and transcripts. The digital recordings were cross-checked with the interview transcripts that were collated using corresponding database entries and converted to individual word document transcripts at completion of the *diamond* study. Transcripts were available for all participants, and digital recordings were available for 128 CATIs from 85 participants. Two additional questions were found to contain information regarding antidepressant medication use (see questions 3 and 4 in Table 1).

**Figure 1.**
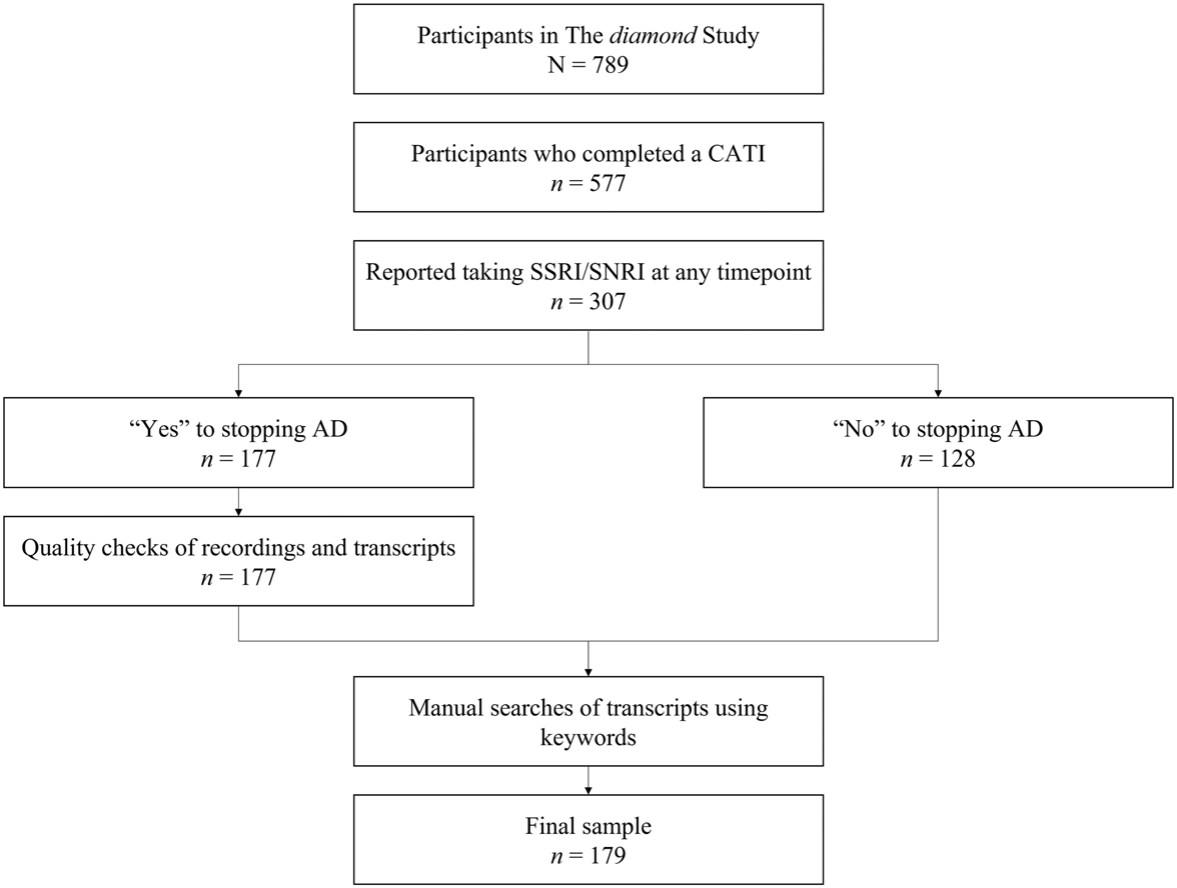
Arriving at the final sample of 179 antidepressant users

AC also conducted manual searches of the transcripts using the keywords ‘antidepressant’ and ‘medication’ for every participant at each time point. No additional information was found for ‘medication’; however, the search for ‘antidepressant’ resulted in two participants being added to the final sample (see Figure1). The findings of the digital recording check and the keyword search were discussed between AC, CKH and JG.

Using a collective case study approach, participant ‘accounts’ of reducing or stopping antidepressants were constructed. Collective case studies are made up of more than one case where information may or may not have been collected at the same time or location. Relevant information collected at each time point or location is then ‘bound’ together to create full case studies for each participant (Mills *et al*., 2010a; 2010b). Collective case studies are useful for establishing an in-depth understanding of an issue in its real-world context (Crowe *et al*., 2011). AC extracted whole responses from question 1 and 2. For questions 3 and 4, only data that related to the deprescribing of antidepressants were extracted. Extracted data were then compiled into individual participant ‘accounts’ (see Box 1 for an example of a participant account after data extractions).

Box 1. Example of a participant account who gave reasons for reducing or stopping antidepressants at multiple time points.

I sort of, I don’t need, I thought I didn’t need it and so stopped taking them for 2 or 3 days and got very depressed and teary (CATI 2) I just thought I’d see how I went not taking it, but it didn’t work out… (CATI 4) I ran out for three days… (CATI 5) I ran out – but probably only about twice (CATI 6) I’ve ran out and I’m a long way from town and just to run 10k into town is a long way… (CATI 9) I ran out and I had no need to go into town. Maybe I didn’t have enough money, I don’t know. It was only maybe three days… (CATI 10)

### Data analysis

Once the accounts had been collated, they were discussed by AC, CKH and JG. Each team member had access to the accounts, audio recordings and original transcripts to confirm accuracy of the final participant accounts. When the research team were satisfied with the participant accounts, they were uploaded into NVivo 12 (QSR International Pty Ltd, 2018) computer software for data analysis and examined for themes and subthemes.

Data analysis was led by AC who was guided by Braun and Clarke’s reflexive thematic analysis (Braun and Clarke, 2019, 2006). Reflexive thematic analysis is a widely used method for systematically identifying, organising and reporting patterns within qualitative data (Braun and Clarke, 2006). An inductive (data-driven) approach was used rather than applying an existing or pre-conceived analysis framework. Each line of data was subject to coding. AC read and re-read each account and took note of any patterns in the data. Patterns were discussed between AC, CKH and JG and formed the basis for initial coding. A coding framework was developed iteratively and recorded in a codebook. Additional codes were included as the analysis progressed and the framework was altered accordingly. Ten percent of the accounts (*n* = 18) were independently coded by both AC and CKH. No coding discrepancies were found between the authors. From the coding, potential themes were proposed by AC and discussed and approved by AC, CKH and JG. Once coding and themes were finalised, frequencies were calculated allowing for the determination of how often a particular theme was referenced.

#### Ethical considerations

*diamond* was approved by the Human Research Ethics Committee at the University of Melbourne (HREC ID: 030613X). Informed, written consent was obtained from all participants via post. Prior to the current authors receiving the data, all data had been deidentified, including any person identified in interviews that was not a participant in the study.

## Results

Of the 307 participants, 179 (58%) reported reducing or stopping their antidepressant medication, while 128 (42%) remained on their medication for the duration of the *diamond* study (see Appendix B for full breakdown of respondents at each time point). Demographic characteristics of the participants are summarised in Table 2. Participants who continued or reduced or stopped antidepressants were similar in age, education level and employment status. The majority of participants within both cohorts was female. Those who reported reducing or stopping indicated more severe depressive symptoms at baseline than those who did not stop. However, of those who did reduce or stop, 37% (*n* = 66) reported having no or mild depressive symptoms. For antidepressant users who did not reduce or stop, almost half (*n* = 58; 45.3%) reported having no or mild depressive symptoms. Participants who reported stopping their medication were more likely to have taken it for a longer duration than those who remained on their medication.

**Table 2.** Demographic characteristics for participants who reduced or stopped antidepressants and participants who remained on antidepressants

		Stopped ADM *n* = 179	Did not stop ADM *n* = 128
Age (years), *M (SD)*		46.51 (12.85)	45.79 (12.85)
		*n* (%)	*n* (%)
Sex (female)		131 (73.18)	107 (83.59)
Highest education level reached			
	Completed year 12 or less	95 (53.0)	71 (55.50)
	Certificate/diploma	43 (24.0)	36 (28.10)
	Bachelor’s degree or higher	41 (23.0)	21 (16.40)
Employment status			
	Full-time	42 (23.46)	29 (22.66)
	Part-time/casual	49 (27.37)	27 (21.09)
	Full-time education	14 (7.82)	13 (10.16)
	Unemployed, looking for work	13 (7.26)	10 (7.81)
	Unable to work due to sickness or disability	28 (15.64)	24 (18.75)
Length of ADM treatment*			
	<1 month to <3 months	25 (15.26)	27 (21.43)
	3 months to <1 year	17 (10.35)	31 (24.60)
	1 year to 2+ years	121 (73.78)	68 (53.97)
	Don’t know	1 (0.61)	–
Depression severity (PHQ-9**), *M (SD)*		12.65 (6.27)	11.71 (6.40)
	None-minimal	14 (7.8)	16 (12.5)
	Mild	52 (29.1)	42 (32.8)
	Moderate	49 (27.4)	29 (22.7)
	Moderately severe	29 (16.2)	22 (17.2)
	Severe	35 (19.5)	19 (14.8)

Note: ADM = antidepressant medication; *n variation due to missing data; ** PHQ-9 = Patient Health Questionnaire 9-item (Kroenke *et al*., 2001).

### Number of reasons given and CATIs completed

Overall, 179 participants gave a total of 377 reasons for reducing or stopping their antidepressants. Eighty-four people gave a single reason and 95 people gave multiple reasons (ranging from a total of 2 to 11 reasons either at one CATI time point or across multiple time points; see Appendix C). Almost half of the participants (*n* = 80; 45%) completed CATIs at all 10 time points. However, none of the 80 participants reported a reason for reducing or stopping at every CATI (see Appendix D) due to participants not reducing or stopping or having not been on antidepressant medication in the period prior to the CATI. For participants that gave responses across multiple time points (*n* = 70; 39%), 20 reported the same reason each time; however, no overall trends or patterns related to the overarching themes for reducing or stopping their medication.

### Reasons people reduce or stop their antidepressant medications

Participants reported a wide range of reasons for reducing or stopping their antidepressant medications, which can be encapsulated by five overarching themes: 1. Depression; 2. Medication; 3. Healthcare system; 4. Psychosocial and 5. Financial. Themes and subthemes are detailed below.

#### Depression

Reasons related to the experience of depressive symptoms (or lack thereof) were the most frequently reported reasons (*n* = 96; 53.6%) for reducing or stopping antidepressant medication.

### Severity of depressive symptoms

Many participants (*n* = 74) reported that they were ‘feeling well’ enough to stop their antidepressants. Some attributed this to changes in personal circumstances or the occurrence of a significantly positive life event with one participant stating:

*‘My situation changed, I guess the pressure that I was under with my ex-partner, going through a court change, so… and he stopped harassing me, so I assumed I didn’t need it anymore, because I was feeling things were easier for me’.*

In contrast, some participants (*n* = 7) reported that their depressive symptoms were severe, and they could not find the energy or motivation to take their medication. One participant said:

*‘[I had a] really bad depressive time, I didn’t take anything at all, I didn’t even eat’.*

Two participants reported that they often stopped and started their medication based on the severity of their depressive symptoms, on an ‘as needed’ basis, for example:

*‘I took them one a night. But now I just, er, um, maybe one a week. Well, yeah, as, as I require it, yeah, yeah. I don’t feel like I need these things like I used to’.*

### Testing the water

For 31 participants, feeling well led to attempts to try and manage their depression without the medication for short periods of time. Feelings of wellness also caused others (*n* = 9) to ‘test’ whether their condition had in fact improved or if the alleviation of symptoms was due to the effectiveness of the medication with a participant responding:

*‘Because I thought I was better and I wanted to see whether it was the drugs or me’.*

#### Medication

Almost half (*n* = 74; 41%) of participants reported reasons relating to the medication, influenced their antidepressant use.

### Effects of the medication

Sixty-one participants had experienced adverse side effects from taking antidepressants, causing them to stop treatment. Side effects included weight gain, lethargy, gastrointestinal complaints, light headedness and sexual dysfunction. Some participants mentioned a loss of clarity of thought, for example:

*‘I’m no longer taking them. I was sick of being a space cadet, being lost in my own self, I’d start picking up in the afternoon before I had my next tablet, and I always felt sluggish’.*

Six participants decided to stop their medication because they were concerned about adverse effects when pregnant or breast-feeding. One participant stated:

*‘I was suppose to [take the antidepressants] but I just didn’t want to when I was pregnant’.*

### Medication regimen

Twenty participants reported experiencing disruptions to their medication regimen. Some (*n* = 3) were required to stop their medication due to an upcoming hospital admission which required them to cease any medications. For example one participant said:

*‘I had to stop for a week once because I was having an operation, yeah I had to stop it for week beforehand’.*

Four participants had acute illnesses such as a throat infection or gastroenteritis where they found taking medication intolerable. Some participants (*n* = 6) reported that their medication regimen was burdensome (‘pill burden’) which led to them to skip dosages or completely stop, as demonstrated by a participant who said:

*‘Keeping the energy to keep on top of it and it’s another tablet – coz I’ve gotta take all these other tablets for my back and my neck, it’s another tablet and I feel a junkie’.*

#### Healthcare

Sixty-three (35.2%) participants reported reasons relating to their healthcare as having an influence on their decision to reduce or stop their antidepressants.

### Clinician input and relationship with patient

Thirty participants mentioned that they had received advice from their clinician to reduce or stop. For 22 participants this was a GP, for four a psychiatrist and for one a Chinese medicine practitioner. Six participants reported initiating the deprescribing discussion with their healthcare provider. One participant said:

*‘I did mention it to the doctor and asked “what about cutting back on the Effexor” and he said I could try and it and let him know’.*

Eight participants indicated that there was a lack of understanding between themselves and their clinician where they were unhappy with the care or diagnosis of depression (where depressive symptoms may have been present but participant did not consider it to be the root problem), and this led them to stop their medication. For example:

*‘When I was first diagnosed with depression, you go through a series of questionnaires with the doctor. When you go back to the doctor because you’re feeling depressed, and you might use that word, you’re no longer asked how far-reaching that is in your life… You’re re-medicated, which I have a problem with. I don’t think that that is constructive, because it doesn’t actually seek out the nature of depression’.*

### Clinician and medication accessibility

Twenty-four participants reported that they ‘ran out’ of the medication prior to filling a new prescription. Five participants reported being unable to access their prescribing clinician when they required a new prescription, one participant had to wait for their pharmacy to order and receive the medication, and two participants were too emotionally unwell to visit the pharmacist. This caused a break in treatment until they were able to receive their medication. However, two participants noted that they used running out as a reason for stopping permanently.

#### Psychosocial

Thirty-five (19.5%) participants reported cognitive, psychological and social reasons for stopping their antidepressants. A desire to recover from depression without the aid of medication was a recurring narrative throughout this theme. The perceived addictive nature of antidepressants and other long-term effects of the medication were of concern to nine participants leading them to stop treatment. One participant said:

*‘I was concerned about long term effect and didn’t want to be always relying on medication’.*

Nineteen participants perceived antidepressants as ‘controlling’ forces on their emotions which was troubling for participants who expressed a desire to recover from depression on their own. Seven participants reported perceptions of self-stigma as a reason to stop their medication with one participant reporting that:

*‘I feel that if I have to take medication it’s a sign of weakness and sometimes I feel that it’s better not to take it and face the problem and deal with it directly’.*

#### Financial

Fourteen (7.8%) participants reported that they could not afford their medication. For some (*n* = 10), this meant that they had to wait until payday to renew their prescription. However, others (*n* = 4) found that the cost of the medication meant that they could no longer afford continue treatment as suggested by a participant who said:

*‘Um, they were getting too expensive. They weren’t on the PBS and I was paying a lot for them and I couldn’t afford it, because I’m on the pension’.*

**Figure 2.**
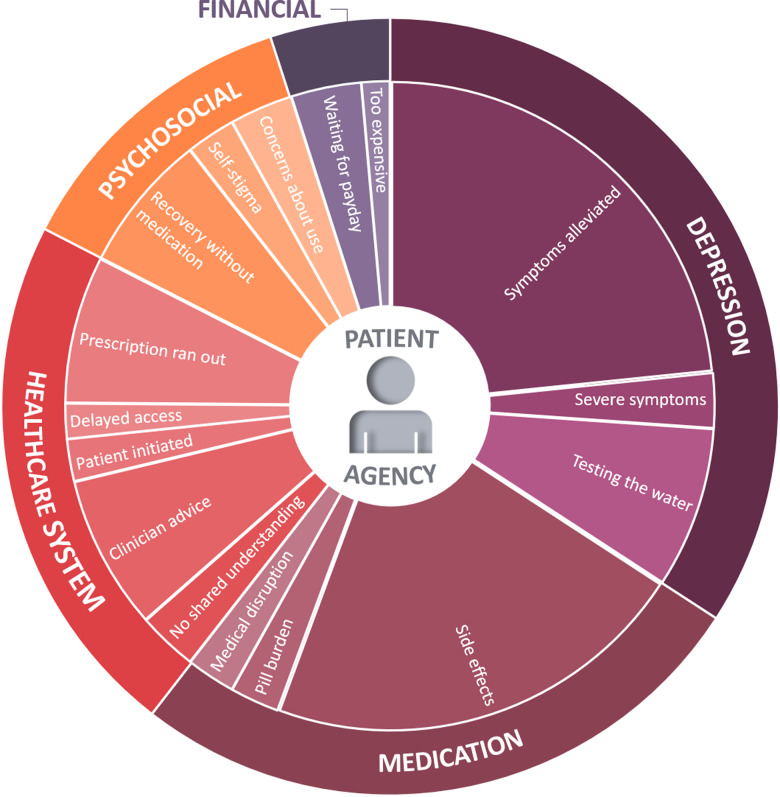
Themes and subthemes for the self-reported reasons general practice patients have for reducing or stopping their antidepressant medications

Figure 2 shows a visual summary of the reasons that influence people to reduce or stop their antidepressants. The figure has been arranged to indicate the frequency of each theme (outer ring) and subtheme (inner ring). As these factors influence patient decision-making, patients have been placed at the centre of the five identified themes. Patient agency represents patients who are reducing or stopping with or without guidance are making and acting on decisions about their own healthcare (Bok *et al*., 2022).

## Discussion

The current study found that primary care patients give a variety of reasons for reducing or stopping their medication that can be summarised in five themes: 1. Depression; 2. Medication; 3. Healthcare system; 4. Psychosocial and 5. Financial.

The findings are consistent with patient experience studies from the UK and the Netherlands where the perceived addictive nature of antidepressants and stigma and a desire to manage their depression without medication were found to be reasons to stop antidepressant treatment. Echoing responses from UK antidepressant users (Leydon *et al*., 2007), patients in the current study also reported that feeling better led them to question how much these feelings of wellness were a true improvement of their depression or if it was the medication. However, where participants in previous studies continued their medication in the face of uncertainty, many of those in the current study opted to ‘test’ the degree of improvement by stopping their medication.

Consistent with past research (Leydon *et al*., 2007; Read *et al*., 2019; Verbeek-Heida and Mathot, 2006), when a GP or psychiatrist was involved in the decision to reduce or stop, participants were willing to deprescribe their medications. However, a clinician was only involved in the decision to stop for 16% (*n* = 30) of participants and most participants reported that they initiated the deprescribing discussion. An emerging element of the doctor–patient relationship that influenced stopping was also seen when participants held negative perceptions of the clinical care they were receiving, particularly when the clinician was seen to provide prescriptions without determining the need for continued treatment. Recently, depression treatment guidelines in the UK (National Institute for Health and Care Excellence (NICE), 2022) and Australia (Malhi *et al*., 2021) have been updated to include information about the benefits and risks of antidepressants, expected duration of treatment and guidance for stopping. Previously, GPs have reported being unaware of official guidelines for antidepressant deprescribing. Therefore, it is important to ensure that such guidance is provided to GPs to support implementation of deprescribing in practice and enhance GP and patient knowledge and decision-making and potentially strengthen GP–patient relationships.

Previous qualitative studies of patient experiences (Leydon *et al*., 2007; Verbeek-Heida and Mathot, 2006) have not reported on the side effects of the antidepressant medication itself, which were commonly reported as reasons for stopping treatment in this study. Experiencing side effects has been shown qualitatively as a factor for stopping antidepressant in medication adherence studies (Zhu *et al*., 2020) and community surveys of patient experiences of antidepressant use (Read *et al*., 2019). A systematic review of qualitative studies also found side effects to facilitate patient willingness to discontinue (Maund *et al*., 2019a). One explanation for this could be that side effects in the early stages of antidepressant treatment are common, and the qualitative studies presented here focused on participants with long-term antidepressant use. However, the findings of the current suggest that side effects do continue after the initial stages of treatment.

The unaffordability of antidepressants and access to healthcare were also new findings. Twenty-three per cent of participants in this study were unemployed or unable to work due to illness which may have impacted the affordability of their medications. Additionally, the differences in funding for antidepressant prescriptions in other countries may indicate why financial reasons were not seen in the qualitative studies from the UK and Netherlands. The majority of SSRI and SNRI medications in Australia are available under the government Pharmaceutical Benefits Scheme (PBS) with out-of-pocket costs varying from AUD$6.80 to AUD$42.50. In 2019–20, 61.7% of mental health-related prescriptions were subsidised under the PBS (Australian Institute of Health and Welfare, 2022), but it has been suggested that PBS medication costs are still high, particularly for people with chronic illness (Duckett and Banerjee, 2017). Comparatively, medications are a flat cost of GPB₤9.35 (approx. AUD$16.52) or are free for eligible people (e.g., people who require income assistance, are on jobseeker, are over 60 years or under the age of 16 years) within the UK National Health Service (National Health Service, 2020). In the Netherlands, residents are required to have health insurance, under which the cost of medications are largely covered (depending on the health insurer) and GP and primary mental healthcare are free (Ministry of Health, Welfare and Sport, 2015).

### Strengths and limitations

The data analysed in this study were collected as part of interviews where the focus was not solely on antidepressant use. Therefore, responses were brief and, at times, lacking in narrative or context. Additionally, not every participant completed an interview at each time point. A collective case study approach allowed us to target, collate and create participants accounts from relevant responses across and within data collection time points and focus on whole participant accounts of stopping antidepressants during analysis. However the nature of the data also meant that any quantitative patterns across the time points were unable to be analysed in the current study but would be of interest for future studies.

There are several strengths that the data offered in this study: 1. The sample size of 179 participants is considered large for qualitative analysis (Fugard and Potts, 2015); 2. Participants came from diverse backgrounds with differing experiences of depression care across a large number of general practices. Analysis of *diamond* participants has shown no to small demographic differences between those who participated in the study and those who did not, indicating that the *diamond* cohort is a representative sample of primary care patients with depressive symptoms (Gunn *et al*., 2008), and; 3. The 10-year data collection period and the emergence of new reasons suggests that the study has potentially captured most of the reasons people have for reducing or stopping their antidepressants.

### Clinical implications

The findings of the study place importance on the initial deprescribing discussion between clinicians and patients. Many participants reported feeling well enough to stop antidepressant treatment, but only a smaller number of people stopped in conjunction with their clinician. Unsupervised antidepressant cessation has been shown to increase the likelihood of withdrawal effects and restarting of medication (Leydon *et al*., 2007; van Geffen *et al*., 2011). Routine deprescribing discussions may allow clinicians to identify patients who are thinking about stopping before they actually do stop without support. Routine deprescribing conversations could also improve prescribing and deprescribing within treatment guidelines. In this study, 63% of people who had reduced or stopped had moderate to severe depressive symptoms indicating that continuation of their antidepressant would have been appropriate. However, for antidepressant users who did not reduce or stop, almost half (46%) reported no to mild depressive symptoms indicating that they may have been ready to deprescribe.

The deprescribing discussion may also help to address concerns, stigma and beliefs around antidepressant use and provide opportunities for patients to be referred to appropriate psychological therapies to support their emotional well-being. Psychological therapies have been shown to be effective in treating depression when used in conjunction with antidepressant treatment (Cuijpers *et al*., 2014) and also when engaging in deprescribing (Maund *et al*., 2019b). Finally, for patients who report medication or financial reasons may benefit from additional support around medication adherence. Though the focus may shift from deprescribing to adherence, a routine discussion may allow the clinician to become aware of the issues with adhering to treatment. Similarly, discussions could address prescription renewals (both the continued need for or addressing the issue of running out) before the next prescription is required to be issued or filled.

One way to ensure that the discussion is initiated, identification of patients who have been emotionally stable and taking antidepressants for >12 months via electronic medical record alerts may be one option for prompting the deprescribing discussion where clinicians can gather information about patient antidepressant use (Coe *et al*., 2021; Isenor *et al*., 2020). Patient agency over their own healthcare should also be considered. Patients may benefit from receiving information about their antidepressant medication at the time of initial prescription which informs them of the possibility of revisiting treatment in the future. Awareness of the potential of deprescribing discussions could empower patients to broach the topic with their clinician, rather than stopping on their own.

## Conclusion

Patients do reduce or stop their antidepressant medications without professional guidance for a variety of reasons. The current study adds support to the literature that suggests that GP or clinician input into the deprescribing decision is important. The implementation of routine deprescribing discussions into clinical practice may support trust building and shared decision-making between patients and clinicians. These deprescribing discussions could be key to reducing long-term and unnecessary antidepressant treatment.

## Supplementary material

For supplementary material accompanying this paper visit http://doi.org/10.1017/S1463423623000038. click here to view supplementary material
