# Supplementary material for: Self-reported reasons for reducing or stopping antidepressant medications in primary care: thematic analysis of the diamond longitudinal study
Source: Prim Health Care Res Dev. 2023 Feb 27;24:e16. doi: 10.1017/S1463423623000038 (PMC9972355; doi:10.1017/S1463423623000038)
Supplement: Supplementary file 1 [file S1463423623000038sup001.docx]

**Appendices**

***Appendix A.*** *diamond* CATI questions with participant responses to stopping antidepressant medications.

Note: * indicates revised question for future timepoints

|  | Question | CATI Timepoint | | | | | | | | | | |
| --- | --- | --- | --- | --- | --- | --- | --- | --- | --- | --- | --- | --- |
|  |  | 2  (12m) | 3  (24m) | 4  (36m) | 5  (48m) | 6  (60m) | 7  (72m) | 8  (84m) | 9  (96m) | 10 (108m) | 11 (120m) |  |
| 1 | We find a lot of people stop taking their medication from time to time. Since you were first prescribed medication for your emotional well-being have you ever stopped taking any of these? (Yes/No). What made you stop (text response) | ✓ |  |  |  |  |  |  |  |  |  |  |
|  | We find that a lot people stop taking their medication from time to time. In the past 12 months, have you stopped taking [antidepressant medication name] even if only for a short time? (Yes/No) What made you stop taking [antidepressant medication name]? (text response)* |  | ✓ | ✓ | ✓ | ✓ | ✓ | ✓ | ✓ | ✓ | ✓ |  |
| 2 | Of all that you have done/tried [for your depression, stress or worries], what was the least helpful? (text response) Why? (text response) | ✓ |  |  |  |  |  |  |  |  |  |  |
|  | During the past 12 months, what has help you least of all with depression stress or worries? (text response)* |  | ✓ | ✓ |  |  |  |  |  |  |  |  |
|  | During the past 12 months, of everything you have done or tried for depression, stress or worries, what has been the least helpful? (text response)* |  |  |  | ✓ | ✓ | ✓ | ✓ | ✓ | ✓ | ✓ |  |
| 3 | Are you for some reason not taking any (other) medicines which have been prescribed for you (that you are supposed to be taking) for your emotional or physical well-being? (Yes/No) Why are not taking them? (text response) |  | ✓ | ✓ | ✓ | ✓ | ✓ | ✓ | ✓ | ✓ | ✓ |  |
| 4 | How have you been feeling since we last spoke about 12 months ago? (text response) |  | ✓ | ✓ |  |  |  |  |  |  |  |  |
|  | Can you describe how you have been feeling since we last spoke about 12 months ago? (text response)* |  |  |  | ✓ | ✓ | ✓ | ✓ | ✓ | ✓ | ✓ |  |

***Appendix B.*** Breakdown of CATI respondents who stopped antidepressants at each timepoint.


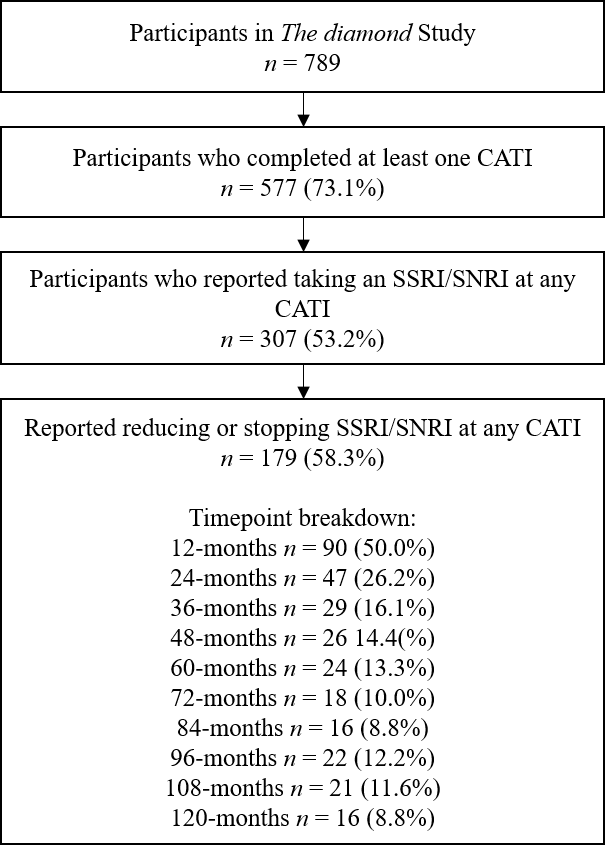


***Appendix C.*** Number of reasons given by number of participants.

***Appendix D.*** Number of CATIs completed and number of CATIs where participants gave a reason for reducing or stopping antidepressant medication, by number of participants.
